# Early Postnatal Manganese Exposure Causes Lasting Impairment of Selective and Focused Attention and Arousal Regulation in Adult Rats

**DOI:** 10.1289/EHP258

**Published:** 2016-07-06

**Authors:** Stephane A. Beaudin, Barbara J. Strupp, Myla Strawderman, Donald R. Smith

**Affiliations:** 1 Department of Microbiology and Environmental Toxicology, University of California, Santa Cruz, Santa Cruz, California, USA; 2 Division of Nutritional Sciences, and; 3 Department of Psychology, Cornell University, Ithaca, New York, USA

## Abstract

**Background:**

Studies in children and adolescents have associated early developmental manganese (Mn) exposure with inattention, impulsivity, hyperactivity, and oppositional behaviors, but causal inferences are precluded by the correlational nature of the data and generally limited control for potential confounders.

**Objectives:**

To determine whether early postnatal oral Mn exposure causes lasting attentional and impulse control deficits in adulthood, and whether continued lifelong Mn exposure exacerbates these effects, using a rat model of environmental Mn exposure.

**Methods:**

Neonates were exposed orally to 0, 25 or 50 mg Mn/kg/day during early postnatal life (PND 1–21) or throughout life from PND 1 until the end of the study. In adulthood, the animals were tested on a series of learning and attention tasks using the five-choice serial reaction time task.

**Results:**

Early postnatal Mn exposure caused lasting attentional dysfunction due to impairments in attentional preparedness, selective attention, and arousal regulation, whereas associative ability (learning) and impulse control were spared. The presence and severity of these deficits varied with the dose and duration of Mn exposure.

**Conclusions:**

This study is the first to show that developmental Mn exposure can cause lasting impairments in focused and selective attention and arousal regulation, and to identify the specific nature of the impairments. Given the importance of attention and arousal regulation in cognitive functioning, these findings substantiate concerns about the adverse effects of developmental Mn exposure in humans.

**Citation:**

Beaudin SA, Strupp BJ, Strawderman M, Smith DR. 2017. Early postnatal manganese exposure causes lasting impairment of selective and focused attention and arousal regulation in adult rats. Environ Health Perspect 125:230–237; http://dx.doi.org/10.1289/EHP258

## Introduction

Elevated environmental manganese (Mn) exposure is emerging as a significant public health problem in the United States and elsewhere, where vulnerable children may be exposed to elevated levels of Mn from drinking water (Bouchard et al. 2011; Ljung and Vahter 2007), soil and dust (Lucas et al. 2015; Lucchini et al. 2012), and their diet (Crinella 2012). Studies of children and adolescents have linked developmental environmental Mn exposure to inattention, impulsivity, hyperactivity, oppositional behaviors, and impaired fine motor function (Bhang et al. 2013; Ericson et al. 2007; Lucchini et al. 2012; Oulhote et al. 2014; Takser et al. 2003), but these studies are limited by their cross-sectional designs and limited control of confounding, making it impossible to infer that Mn causes these impairments. In addition, these studies have used behavioral measures that do not allow delineation of the specific functional deficits that underlie the poorer performance of the Mn-exposed children. Animal studies have demonstrated that early postnatal Mn exposure can impair performance on tests of learning and memory and motor function (Golub et al. 2005; Kern et al. 2010; Reichel et al. 2006), but none have provided assessments of attentional function to inform interpretation of the observational human findings.

Attentional dysfunction, including attention deficit hyperactivity disorder (ADHD), is the most prevalent neurodevelopmental disorder among children, affecting ~ 6–11% of all U.S. children between 6 and 17 years of age, with two to three times as many males affected as females (Feldman and Reiff 2014; Willcutt 2012). Although the etiology of attentional deficits and ADHD remains unclear, it is clearly multifactorial. Neuropsychological and imaging studies in children have shown that ADHD (and attentional dysfunction more broadly) is generally associated with hypofunctioning of catecholaminergic systems within the cortico-striatal loop (Arnsten 2010; Brennan and Arnsten 2008). In light of these data, it is noteworthy that studies in animal models have shown that early postnatal Mn exposure alters catecholamine function in these same brain areas (Kern and Smith 2011; Kern et al. 2010; McDougall et al. 2008; Reichel et al. 2006). Delineating the specific functional impairments produced by potential neurotoxicants such as Mn, and elucidating their neural bases is key to devising effective treatment and prevention strategies.

In the present study we used a rodent model of early childhood oral Mn exposure to determine whether Mn causes enduring impairments in focused and selective attention, impulse control, and associative ability (learning), using a series of tasks that are variants of the five-choice serial reaction time task (5-CSRTT). The attention tasks are well-accepted animal homologues of clinical tests used to assess attentional function in children and adults (Bari et al. 2008; Robbins 2002). Given the emerging evidence that Mn exposure history may be associated with adverse neurobehavioral effects in infants and children in a non-linear fashion (Bhang et al. 2013; Claus Henn et al. 2010; Lucchini et al. 2012; Oulhote et al. 2014; Takser et al. 2003), we also tested whether continued oral Mn exposure throughout postnatal life exacerbated the effects of the early postnatal exposure. Our findings are the first to show that developmental Mn exposure can cause lasting impairments in attention and arousal regulation, supporting the reported associations between Mn exposure and deficits in these functional areas in children.

## Materials and Methods

### Subjects

A total of 115 Long-Evans male rats were used for neurobehavioral assessment. Additional littermates were used for tissue Mn analysis. All subjects were born in-house from 24 nulliparous timed-pregnant rats (Charles River; gestational age 18). Twelve to 24 hours after parturition (designated PND 1, birth = PND 0), litters were sexed, weighed, and culled to eight pups per litter such that each litter was composed of five to six males and the remainder females. Only one male per litter was assigned to a particular treatment condition, with *n* = 21–23 animals per treatment group. Animals (dams and weaned pups) were fed Harlan Teklad rodent chow #2018 (reported by the manufacturer to contain 118 mg Mn/kg) and housed in polycarbonate cages at a constant temperature of 21 ± 2°C. At PND 22, all pups were weaned and pair-housed (two rats per cage) with an animal of the same treatment group and maintained on a reversed 10:14 hr light/dark cycle. All aspects of testing and feeding were carried out during the active (dark) phase of the animals’ diurnal cycle. Males were used because human and animal studies have shown that males are more sensitive than females to developmental Mn neurotoxicity (Kern et al. 2010; Lucchini et al. 2012; Takser et al. 2003), and attentional dysfunction is two to three times more prevalent in boys than girls (Feldman and Reiff 2014; Willcutt 2012). All animal care and treatments were approved by the institutional IACUC and adhered to National Institutes of Health guidelines set forth in the Guide for the Care and Use of Laboratory Animals.

### Manganese Exposure

Neonates were orally exposed to 0, 25, or 50 mg Mn/kg/day from either PND 1–21, or PND 1 until the end of the study (~ PND 192). For dosing over PND 1–21, Mn was delivered once daily directly into the mouth of each pup (~ 25 μL/dose) via a micropipette fitted with a flexible polyethylene pipet tip. Control animals received only the vehicle solution (see Supplemental Material, “Manganese exposure protocol and rationale”). After weaning starting on PND 22, Mn was administered via the drinking water at levels of ~ 210 μg Mn/mL or ~ 420 μg Mn/mL for the 25 or 50 mg Mn/kg/day exposure groups, respectively; actual water Mn levels were adjusted weekly if needed to maintain target exposure levels based on water intake. Water bottle weights were recorded at refilling to determine water intake per cage, and daily Mn intake per kg body weight was estimated based on daily measured body weights of the two post-weaned rats housed per cage. These Mn exposure regimens are relevant to children exposed to elevated Mn via drinking water, diet, or both; pre-weaning exposure to 25 and 50 mg Mn/kg/day produces relative increases in Mn intake that approximate the increases reported in infants and young children exposed to Mn-contaminated water or soy-based formulas (or both) (Kern et al. 2010). Chronic oral exposure to the same Mn doses were maintained after weaning via drinking water, since children may continue to suffer chronic elevated Mn exposures from a variety of environmental sources (e.g., contaminated well water, dust, etc.) (Bouchard et al. 2011; Lucas et al. 2015; Oulhote et al. 2014) (see Supplemental Material, “Manganese exposure protocol and rationale” for more information on the environmental childhood relevance of these exposure regimens).

### Testing Apparatus

Eight identical automated 5-CSRTT testing chambers fitted with odor delivery systems (#MED-NP5L-OLF, Med Associates, Inc., St. Albans, VT) were used to assess specific cognitive processes, including focused and selective attention and inhibitory control, as described previously (Stangle et al. 2007). Briefly, each testing chamber contained a curved aluminum wall equipped with five 2.5 × 2.5 cm response ports positioned 2 cm above the grid floor; each port was fitted with a light-emitting diode that served as the visual cue, an infrared beam to register nose pokes, and pneumatic inlet and vacuum outlet ports to introduce and remove air-based odor distractors. Opposite the response wall was the food magazine wall that contained a 45 mg food pellet dispensing port fitted with an infrared beam to register nose pokes. The two side walls and ceiling were polycarbonate, and the floor was a grid of stainless steel rods; each unit also contained a small house light. The entire testing chamber was enclosed in a sound attenuating cubicle.

### Behavioral Testing

Behavioral testing began on ~ PND 80, with food magazine and nose-poke training for 1 week followed by two 5-choice visual discrimination tasks with a fixed cue duration and no pre-cue delay, and then followed by a series of attention tasks as described below (see Supplemental Material, “Behavioral testing procedures” for details). All rats were weighed and tested 6 days/week throughout training and testing. Behavioral assessment occurred during the active (dark) period of the diurnal cycle at the same time each day and in the same chamber for each individual rat. A daily test session consisted of 120 trials or 60 min, whichever came first. Each trial sequence was initiated by a nose-poke in the food magazine port, and followed by a 3 sec turnaround time to allow the animal to reorient from the food magazine wall to the response wall; trial onset began after the 3 sec turnaround time. All behavioral testing was conducted by individuals blind to the treatment condition of the subjects. All animals were maintained on a food restriction schedule with water available *ad lib* throughout behavioral assessment, as described previously (Beaudin et al. 2013).

### Focused Attention Tasks

Focused attention can be defined as the ability to maintain attentional focus on a specific task or stimulus (e.g., a visual cue). Two focused attention tasks (#1 and #2) were administered over PND 101–121, and PND 122–133, respectively, following completion of the visual discrimination task (see Supplemental Material, “Behavioral testing procedures”). The first focused attention task used variable pre-cue delays of 0, 3, 6, or 9 sec and a fixed visual cue duration of 1 sec and was administered for 20 sessions. The second focused attention task included variable pre-cue delays of 0, 3, or 6 sec and variable visual cue durations of 0.5 or 1.0 sec, and was administered for 12 sessions. Both focused attention tasks assessed the ability of the animals to detect and respond to a brief visual cue presented unpredictably in time and location (one of the five response ports).

### Selective Attention Task with Olfactory Distracters

Selective attention can be defined as the ability to maintain a behavioral or cognitive set in the face of distracting or competing stimuli (Petersen and Posner 2012). The final two tasks administered were the selective attention baseline task and the selective attention task with olfactory distractors. Animals were tested in the selective attention baseline task for three daily test sessions. This task was identical to the preceding focused attention task number 2 except that the pre-cue delay varied between 3 and 4 sec, with the two delays balanced across the trials within each test session. This task was followed by the selective attention task for 12 sessions, which was identical to the baseline task except that on one third of the trials in each session, an olfactory distractor was presented 1 or 2 sec after trial onset (i.e., 1–3 sec before the visual cue). The nine olfactory distractors were made from liquid odorants (McCormick & Company, Inc.) diluted in propylene glycol, and delivered as scented air (see Supplement Material, “Behavioral testing procedures” for details).

Recorded response types for all attention tests included the following: premature responses (responses made after trial onset but before presentation of the visual cue); correct response (responses made to the correct port following presentation of the visual cue); incorrect response (responses made to the incorrect port following presentation of the visual cue); and omissions (failure to respond within the 10 sec response interval following the visual cue). Premature and incorrect responses and omission errors were not rewarded and were immediately followed by a 5 sec time-out, in which the house light was turned off for 5 sec. In addition, the latency for correct responses was recorded, as was the latency to retrieve the food pellet reward following a correct response (see Supplemental Material, “Behavioral testing procedures” for details). The calculated response outcomes were percent correct, calculated as number of correct responses/(correct + incorrect + premature + omissions) × 100; percent incorrect, calculated as above but with incorrect responses in the numerator; percent accuracy, calculated as number of correct responses/(correct + incorrect) × 100; percent premature, calculated as number of premature responses/(correct + incorrect + premature + omissions) × 100; and percent omissions, calculated as number of omissions/(correct + incorrect + premature + omissions) × 100.

### Blood and Brain Mn Levels

Blood and brain Mn concentrations were determined in littermates as well as the study animals at the completion of neurobehavioral testing [~ PND 192, as previously described (Kern et al. 2010; Beaudin et al. 2013)]. Briefly, whole blood was digested using trace metal clean methods and analyzed for Mn by inductively coupled plasma–mass spectrometry (Thermo Element XR). The analytical detection limit for Mn was 0.04 ng/mL (see Supplemental Material, “Methods for determining blood and brain Mn concentrations” for details).

### Statistical Methods

The behavioral data were modeled by way of structured covariance mixed models. Fixed effects included in the model were Mn treatment (five levels corresponding to the five treatment groups), pre-cue delay, cue duration, session block, and/or distraction condition depending on the outcome analyzed. In all models, the random effect was rat to account for correlations within observations from the same animal. Statistical tests used a Satterthwaite correction. Plots of residuals by experimental condition were used to examine the assumption of homogeneity. Additional random effects with high variance in the residuals across the levels of the factor (e.g., distraction condition) were added to achieve homogeneity if needed. The distribution of each random effect was inspected for approximate normality and presence of influential outliers. Blood and brain Mn data were analyzed using a one-way analysis of variance and Tukey’s *post hoc* test for pairwise comparisons.

The significance level was set at *p* ≤ 0.05, and *p*-values between 0.05 and 0.10 were considered to be trends and are presented if the pattern of findings aided in clarifying the nature of the Mn effects. Significant main effects or interaction effects were followed by single-degree of freedom contrasts in order to clarify the nature of the interactions, using the Student’s *t*-test for pairwise comparisons of least squared means. All analyses were conducted using SAS (version 9.4) for Windows on a mainframe computer or JMP (version 11.0; SAS Institute, Inc.).

## Results

There were significant adverse effects of oral Mn exposure on multiple response measures, including percent correct, incorrect, and accurate responses. Although all three of these complementary outcome measures provided compelling evidence for impaired attention, below we focus on the results for response accuracy due to space constraints and because this dependent measure most clearly differentiated the Mn treatment groups from the controls, and delineated the nature of the attentional dysfunction. Findings on the other response measures are presented in full in the Supplemental Material, “Animal body weights over the course of the study” and in “Behavioral testing results augmenting results provided in the main text” and Figures S2 and S3.

### Focused Attention Task

**Figure 1:**
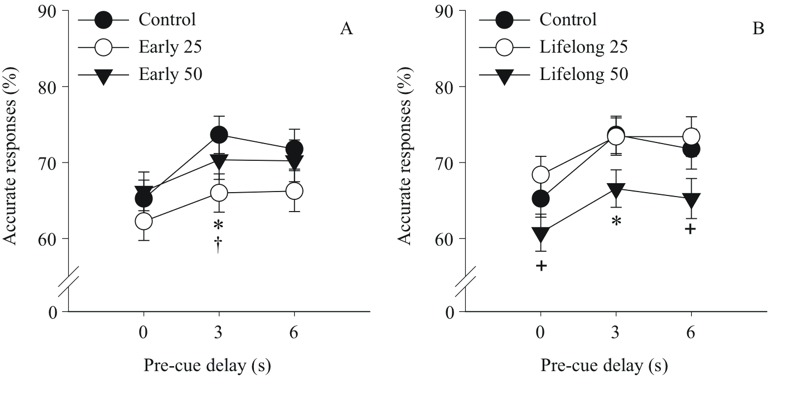
Postnatal Mn exposure causes dose and duration-dependent deficits in the focused attention task. Accurate responses (%) for (*A*) the early postnatal Mn groups and (*B*) the lifelong postnatal Mn groups, as a function of increasing pre-cue delay in seconds (s) (*n *= 21–23/group). *p ≤ 0.05 versus controls. ^+^Significant difference (*p *≤ 0.05) between the 50 versus 25 mg Mn/kg/day groups. ^†^Significant difference (*p *≤ 0.05) between the early 25 group in (*A*) and the lifelong 25 mg Mn/kg/day group in (*B*). Note: The statistical model included all five treatment groups, but results are presented by exposure duration for clarity.

***Postnatal Mn exposure causes dose-dependent deficits in the focused attention task.*** The second focused attention task that included variable pre-cue delays and cue durations uncovered significant adverse effects of the early postnatal Mn exposure on correct and incorrect responses, and response accuracy (Mn × pre-cue delay interaction, percent correct F(8, 391) = 2.07, *p* = 0.037; percent incorrect F(8, 740) = 3.28, *p* = 0.001; percent accuracy F(8, 389) = 2.65, *p* = 0.008). The significant interaction of Mn exposure and pre-cue delay for percent accuracy reflected the findings that the early postnatal 25 group did not differ from controls for trials with a 0 sec or a 6 sec pre-cue delay (*p* = 0.39 and 0.14, respectively), but had significantly lower response accuracy than controls for trials with a 3 sec pre-cue delay (*p* = 0.03) (Figure 1A). A qualitatively similar but non-significant trend was exhibited by the early postnatal 50 group as well (Figure 1A).

The lifelong 50 group achieved similar percent accuracy as controls for trials with a 0 sec pre-cue delay (*p* = 0.19), but exhibited impaired accuracy relative to controls for trials with a 3 sec pre-cue delay (*p* = 0.04), with a trend also seen for trials with a 6 sec pre-cue delay (*p* = 0.08) (Figure 1B). Contrasts between the lifelong Mn groups revealed that the 50 group also had a significantly lower response accuracy than the 25 mg Mn/kg/day group for trials with a 0 sec (*p* = 0.02) and a 6 sec (*p* = 0.02) pre-cue delay, with a similar trend seen for trials with a 3 sec pre-cue delay (*p* = 0.06). The finding that group differences were less significant for the 6 sec delay (than for the 3 sec delay) may be due in part to reduced power to detect a significant difference, given the markedly reduced number of timely response trials at this delay due to the much higher incidence of premature responses (~ 50% for the 6 sec delay vs 25% for the 3 sec delay).

***Focused attention task deficits depend upon the dose and duration of postnatal Mn exposure.*** The effects of Mn exposure on focused attention varied as a function of both the dose and timing/duration of exposure in a non-monotonic fashion, with animals in the early postnatal 25 group exhibiting significantly lower response accuracy than their lifelong 25 Mn-exposed counterparts for trials with a 3 sec pre-cue delay (*p* = 0.03), with trends also seen for trials with a 0 sec or 6 sec pre-cue delay (*p* = 0.08 and 0.06, respectively) (Figure 1A,B). In contrast, there were no significant differences in response accuracy between the early and lifelong postnatal 50 Mn groups for any pre-cue delay condition. However, the fact that the lifelong 50 group differed significantly from controls for the 3 sec pre-cue delay trials, whereas the early 50 group did not, implies that the additional exposure duration may have worsened the effects of the early exposure at this higher Mn dose (Figure 1A,B).

### Selective Attention Task

**Figure 2:**
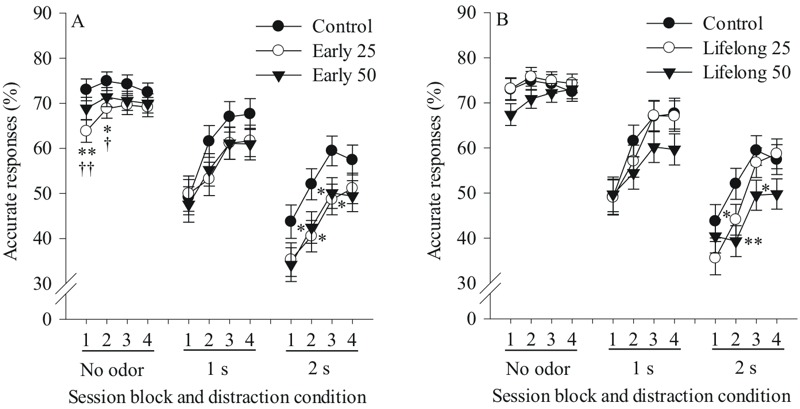
Postnatal Mn exposure causes dose and duration-dependent deficits in the selective attention task. Accurate responses (%) for (*A*) the early postnatal Mn groups and (*B*) the lifelong postnatal Mn groups, as a function of session block for each distraction condition (no distractor, odor distractor 1 second (s) or 2 s into the pre-cue delay interval) (*n* = 21–23/group). * and ** indicate *p *≤ 0.05 and *p *≤ 0.01 versus controls, respectively, and † and †† indicate significant difference (at *p *≤ 0.05 and *p *≤ 0.01, respectively) between the early 25 group in (*A*) and the lifelong 25 mg Mn/kg/day group in (*B*). The statistical model included all five treatment groups, but results are presented by exposure duration for clarity.

***Early postnatal Mn exposure causes lasting deficits in the selective attention task.*** The selective attention task also provided evidence of significant adverse effects of the early postnatal Mn exposure on correct and incorrect responses, and response accuracy [Mn × distracter × session block interaction, percent correct F(24, 2,151) = 1.54, *p* = 0.046; percent accuracy F(24, 2,315) = 1.44, *p* = 0.077; Mn × distracter interaction, percent incorrect F(8, 529) = 2.50, *p* = 0.011]. Although the 3-way interaction for accuracy did not achieve classical significance (*p* = 0.077), the pattern of performance across trial conditions and session blocks suggested several types of attentional impairment that warranted follow up. Broadly speaking, the impairing effect of Mn exposure on response accuracy increased across the three distraction conditions (no distractor, 1 sec, and 2 sec), with the greatest impairment seen for the 2 sec distractor condition (i.e., distractor presented 2 sec into the trial; i.e., 1–2 sec before the visual cue). Specifically, animals exposed to 25 mg Mn/kg/day during early postnatal life had significantly lower response accuracy than controls for the 2 sec distractor condition during session blocks 2 and 3 (*p* = 0.01 and 0.02, respectively), with a similar trend seen during block 1 (*p* = 0.07) (Figure 2A). Response accuracy for this Mn group was also significantly lower than controls for the non-distraction trials during session blocks 1 and 2 (*p* = 0.008 and 0.04, respectively). This group also tended to achieve a lower level of accuracy than controls for the 1 sec distractor condition (distractor presented 1 sec into the trial; i.e., 2 or 3 sec prior to the visual cue) during session block 2 (*p* = 0.10) (Figure 2A).

Similarly, the early postnatal 50 Mn group also exhibited significantly lower response accuracy than controls for the 2 sec distractor condition during session blocks 2 and 3 (both *p*-values = 0.05), with a similar trend during session blocks 1 and 4 (*p* = 0.10 and 0.09, respectively) (Figure 2A). This group, however, was not impaired relative to controls for the non-distraction condition or for the 1 sec distractor condition during any session block (Figure 2A). Moreover, the early postnatal 25 and 50 groups did not significantly differ from each other in response accuracy across any distractor condition and session block (Figure 2A).

There was a significant main effect of distractor condition [F(2, 371) = 341.5, *p* < 0.0001] on response accuracy in the selective attention task, and a significant distractor condition × session block interaction [F(6, 2,315) = 27.98, *p* < 0.0001] (Figure 2). The main effect of distraction condition attests to the disruptive effect of the unpredictable presentation of the olfactory distractors. The interaction of distraction condition and session block reflects the fact that whereas performance was relatively stable across session blocks for the non-distraction trials, performance for the distraction trials significantly improved across sessions, indicating that the disruptive effect of the distractors lessened with extended testing.

***Lifelong postnatal Mn exposure causes deficits in the selective attention task.*** Lifelong postnatal Mn exposure also significantly impaired response accuracy in the selective attention task, most prominently for the lifelong 50 group, who exhibited significantly *lower* response accuracy than controls for the 2 sec distractor condition during session blocks 2 and 3 (*p* = 0.009 and 0.03, respectively), with a similar trend during block 4 (*p* = 0.10) (Figure 2B). The lifelong 50 group also tended to have lower response accuracy than controls for the 1 sec distractor condition during session block 4 (*p* = 0.10), and for trials with no distractor presented during session block 1 only (*p* = 0.10) (Figure 2B). By comparison, accuracy of the lifelong 25 Mn group did not differ from controls for any condition, although a trend towards an effect was seen for the 2 sec distractor condition during session blocks 1 and 2 (both *p*-values = 0.10), with no detrimental effects seen for the 1 sec distractor condition or for the non-distraction condition across session blocks (Figure 2B).

Specific comparison between the lifelong 25 and 50 Mn dose groups shows that the 50 group tended to have a lower response accuracy than their 25 mg Mn/kg/day counterparts for non-distraction trials during session blocks 1 and 2 (both *p*-values = 0.09), and for the 2 sec distractor condition during session block 4 (*p* = 0.059) (Figure 2B).

***Selective attention task deficits depend upon the dose and duration of postnatal Mn exposure.*** Contrasts between the early versus lifelong Mn exposure groups for each dose revealed that the selective attention deficits in adulthood depend upon both the dose and duration of postnatal Mn exposure in a non-monotonic fashion, similar to the effects on focused attention reported above. The early postnatal 25 group exhibited significantly lower response accuracy than their lifelong 25 Mn-exposed counterparts for non-distraction trials during session blocks 1 and 2 (*p* = 0.007 and 0.02, respectively), with similar trends for blocks 3 and 4 (both *p*-values = 0.08), as well as for trials with a 2 sec distractor during session blocks 3 and 4 (*p* = 0.08 and 0.10, respectively) (Figure 2A vs. 2B). In contrast, there were no differences in response accuracy between the early and lifelong 50 mg Mn/kg/day groups for any task condition (Figure 2A vs. 2B).

**Figure 3:**
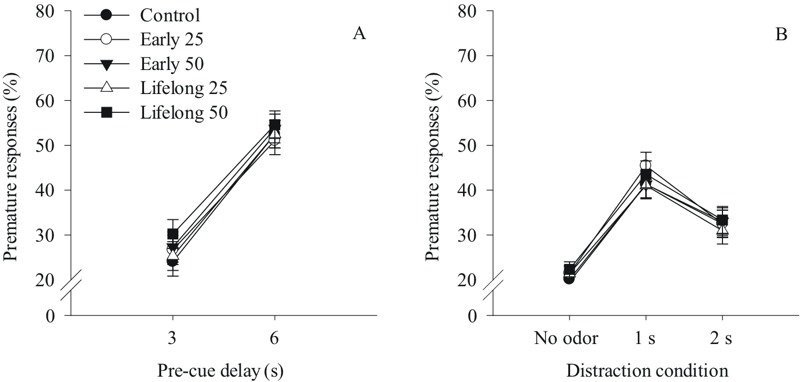
Postnatal Mn exposure did not alter premature responses (%) in (*A*) the focused attention task as a function of increasing pre-cue delay in seconds (s), and in (*B*) the selective attention task as a function of distraction condition (no distractor, odor distractor 1 s or 2 s into the pre-cue delay interval) (*n *= 21–23/group). For (*A*) the 0 s pre-cue delay is omitted because no premature responses are possible at that condition. Oral Mn doses are in mg Mn/kg/day.

***Early or lifelong postnatal Mn exposure does not affect premature responses or omission errors in either the focused or the selective attention tasks.*** There was no effect of Mn exposure on premature responses or omission errors in either the focused attention or selective attention tasks (all *p*-values > 0.5; for premature responses Figure 3A and 3B, respectively). However, in the focused attention task, as the pre-cue delay increased, there was a significant increase in premature responses and a reduction in omission errors for all groups (Figure 3A). Similarly in the selective attention task, the presentation of olfactory distractors significantly increased premature responses in all groups (Figure 3B), but did not alter omission error rates (see Supplemental Material, “Animal body weights over the course of the study” and “Behavioral testing results augmenting results provided in the main text” for detailed results).

***Postnatal Mn exposure produced environmentally relevant body Mn levels, with no effects on body weight or general health.*** Postnatal exposure to 25 or 50 mg Mn/kg/day increased both blood and brain Mn levels in PND 24, 66, and ~ 500 animals in a dose-dependent fashion, though levels were significantly higher in the PND 24 weanlings compared to their older adolescent and adult counterparts; levels in the latter two groups were very comparable to each other and only slightly higher than their age-matched controls (Table 1). All groups gained weight as expected over the course of the study [F(9, 989) = 3,404, *p* < 0.0001], and there was no effect of Mn exposure or an interaction of Mn exposure and age on body weight [F(4, 104) = 0.70, *p* = 0.59 and F(36, 989) = 0.97, *p* = 0.51, respectively] (see Supplemental Material, “Animal body weights over the course of the study” and Figure S1 for more details).

**Table 1:** Blood and brain Mn concentrations in littermates of the behaviorally tested animals (PND 24, 66) and in the behaviorally tested animals at sacrifice (PND ~ 500).

Age (PND)	Control	25 mg Mn/kg/day		50 mg Mn/kg/day	
		Early life	Lifelong	Early life	Lifelong
Blood
24	24.2 ± 0.79 (11)^A,a^	NA	188 ± 28 (17)^B,a^	NA	247 ± 23 (15)^C,a^
66	9.51 ± 0.36 (14)^A,b^	11.8 ± 0.53 (15)^B^	13.7 ± 0.68 (17)^B,^^b^	13.3 ± 0.78 (15)^B^	19.4 ± 1.2 (13)^C,b^
490	5.76 ± 0.28 (16)^A,c^	7.12 ± 0.56 (21)^A^	9.30 ± 0.50 (15)^B,c^	6.83 ± 0.36 (16)^A^	15.2 ± 1.14 (20)^C,b^
Brain
24	3.77 ± 0.19 (11)^A,a^	NA	11.3 ± 2.25 (16)^B,a^	NA	12.8 ± 1.64 (14)^B^^,a^
66	2.12 ± 0.031 (14)^A,b^	2.24 ± 0.037 (14)^A,B^	2.39 ± 0.030 (17)^C,D,b^	2.27 ± 0.041 (16)^B,C^	2.53 ± 0.047 (14)^D,b^
490	1.95 ± 0.063 (13)^A,b^	2.05 ± 0.083 (19)^A,B^	2.24 ± 0.046 (16)^B,b^	1.96 ± 0.051 (12)^A,B^	2.63 ± 0.085 (17)^C,b^
Note: Data are mean ± standard error (*n*); blood Mn in ng/mL, brain Mn in μg/g dry weight. A, B, etc. superscripts: within an age group and tissue, treatment groups with different capital letter superscripts are statistically different from one another (*p* < 0.05), based on Tukey’s *post hoc* test. Lower case a, b, etc.: within a treatment group and tissue, values across ages with different lower case superscripts are statistically different from one another. Main effect statistics are: blood Mn, age F(2,128) = 764, *p* < 0.0001, treatment F(2,128) = 198, *p* < 0.0001, age × treatment F(4,128) = 34.7, *p* < 0.0001; Brain Mn, F(2,123) = 387, *p* < 0.0001, treatment F(2,123) = 89.2, *p* < 0.0001, age × treatment F(4,123) = 20.5, *p* < 0.0001). PND, postnatal day.					

## Discussion

This study is the first to establish that early postnatal Mn exposure can cause lasting attentional dysfunction in a rodent model of childhood Mn exposure, revealing dysfunction in this area that is comparable in magnitude to that exhibited by ADHD children (Bubnik et al. 2015; Wang et al. 2013). In addition, it sheds light on the specific nature of the attention deficits, and the importance of exposure history in causing the dysfunction.

### Selective and Focused Attention Is Impaired by Postnatal Mn Exposure

The Mn-induced dysfunction was most pronounced in the area of selective attention, with all four Mn-exposed groups showing impairment in this cognitive domain. Here, impaired selective attention can be inferred if the disruption in response accuracy produced by the presentation of the olfactory distractor, relative to no distraction, is greater for a given Mn exposure group than for the controls. Such evidence exists for all four Mn exposed groups under the 2 sec distractor condition (Figure 2). Similar patterns were seen for the percent correct and incorrect response measures (see Supplemental Material, “Behavioral testing results augmenting results provided in the main text” and Figure S3). It is noteworthy that the selective attention deficit of the Mn exposed animals emerged largely in the distractor condition that produced the greatest overall disruption in performance; i.e., placed the greatest demand on selective attention ability.

Manganese exposure also impaired performance in the focused attention task, but here the dysfunction was limited to the early postnatal 25 and lifelong 50 Mn groups. As seen in Figure 1, response accuracy for all groups was higher on trials with a 3 sec or a 6 sec pre-cue delay than on trials with a 0 sec pre-cue delay, indicating improved orienting readiness or attentional preparation at the longer delays. However, the early postnatal 25 and lifelong postnatal 50 Mn-exposed groups benefitted less than controls when longer pre-cue delays were given, as indicated by the significant interaction of Mn exposure condition and pre-cue delay. Overall, this pattern of effects suggests that the early postnatal 25 and lifelong postnatal 50 Mn exposure conditions produced impairment in focused attention due to a deficit in preparedness, or the animals’ ability to orient and attend to the impending visual cue (Carli et al. 1983; Totah et al. 2009).

### Impulse Control Is Not Impaired by Postnatal Mn Exposure

The premature response rate provides clear evidence that, for all treatment groups, the long pre-cue delays in the focused attention task challenged inhibitory control, as did the presentation of olfactory distractors in the selective attention task. However, there was no effect of early or lifelong postnatal Mn exposure on the incidence of premature responses for either task (Figure 3A,B). This leads us to conclude that neither early nor lifelong postnatal Mn exposure, at these doses, affects inhibitory control in adulthood. In contrast, others have shown that developmental exposure to other toxicants such as lead and ethanol can impair inhibitory control in rodent models (Sanchez-Roige et al. 2014; Stangle et al. 2007). Our findings of significant attentional deficits due to Mn, without deficits in impulse control shed light on the specific nature of the attention deficits. We should note, however, that the negative findings on impulsivity and association learning reported here do not preclude occurrence of these effects of Mn exposure in children, since the animals reported here were tested as adults.

### Postnatal Mn Exposure Alters Arousal Regulation in Tasks with Unpredictable Trial Conditions

**Figure 4:**
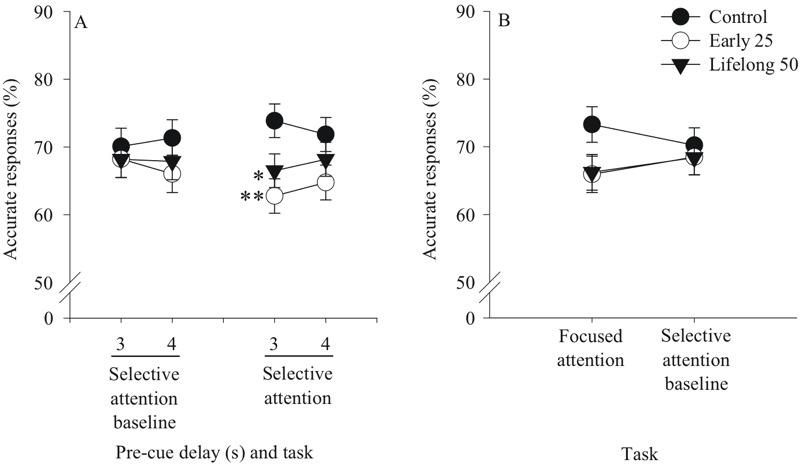
Postnatal Mn exposure altered arousal regulation in tasks with unpredictable trial conditions. Accurate responses (%) for the early postnatal 25 and the lifelong postnatal 50 mg Mn/kg/day dose groups in (*A*) the selective attention baseline and selective attention tasks as a function of pre-cue delay in seconds (s), and (*B*) the focused attention and selective attention baseline tasks (*n *= 21–23/group). For (*A*), performance is shown for the 3-day selective attention baseline task and for the non-distraction trials over the first 3 days (session block 1) of the selective attention task, while for (*B*) performance is shown for all 12 days of the focused attention task and the subsequent 3-day selective attention baseline task (justified by the presence of a higher order interaction involving Mn treatment and session block in the selective attention task, but not the focused attention task). Order of testing was the focused attention task followed by the selective attention baseline task and then the selective attention task. These follow-up statistical models included the fixed effect of Mn treatment, with three levels corresponding to the control, early 25 and lifelong 50 Mn groups. ^*^ and ^**^ indicate *p *≤ 0.05 and *p *≤ 0.01 versus controls, respectively.

Regulation of an optimal arousal state is necessary for many interdependent functions subserved by the prefrontal cortex, including attentional function, planning and decision-making, and behavioral inhibition (Arnsten 2009). Here, both the early postnatal 25 and the lifelong postnatal 50 Mn groups exhibited a transient impairment of response accuracy for non-distraction trials of the selective attention task, being apparent in the first session block of testing but disappearing thereafter. One possible explanation for this transient deficit for the non-distraction trials is that the unpredictable presentation of the olfactory distractors produced a state of over-arousal in these Mn groups over the first several days of testing, which impaired their ability to attend to the visual cue, and that this effect dissipated with further testing. We tested this hypothesis by comparing response accuracy on the non-distraction trials during the first 3-day session block of the selective attention task to performance during the preceding selective attention baseline task, which included identical trial conditions (pre-cue delay, cue duration), but did not include olfactory distractors. Results showed a significant task × Mn treatment × pre-cue delay interaction for response accuracy [F(2, 130) = 4.66, *p* = 0.01], reflecting poorer accuracy for the early 25 and lifelong 50 Mn groups (vs. controls) for trials with a 3 sec pre-cue delay in the selective attention task (*p* = 0.006 and 0.05, respectively), but not for the same pre-cue delay trials of the baseline task (*p* = 0.65 and 0.64, respectively) (Figure 4A). The early postnatal 25 Mn group also showed a trending impairment (vs. controls) in 4 sec pre-cue delay trials of the selective attention task (*p* = 0.08), but not the baseline task (Figure 4A). This pattern of findings indicates that the transient impairment seen for these Mn groups for the non-distraction trials of the selective attention task reflects some characteristic of the selective attention task not seen in the baseline task, such as the unpredictable presentation of olfactory cues, or the fact that task conditions were changing unpredictably from trial to trial in a relatively pronounced way. A likely explanation is that this transient dysfunction seen for these non-distraction trials reflects over-arousal, due to either a more pronounced arousal response or an impaired ability to regulate the heightened arousal (Carli et al. 1983; Raz and Buhle 2006).

A similar analysis was conducted to test whether impaired arousal regulation may have also contributed to the inferior response accuracy of these Mn groups in the 3 sec and 6 sec pre-cue delay trials of the focused attention task. This task may have also engendered heightened arousal due to the unpredictable presentation of long pre-cue delays on some trials (Carli et al. 1983). To test this hypothesis, response accuracy in the 3 sec pre-cue delay trials of the focused attention task was compared to the 3 sec pre-cue delay condition of the selective attention baseline task, which immediately followed the focused attention task. Results revealed a significant task × Mn treatment interaction [F(2, 64) = 4.03, *p* = 0.02], reflecting that response accuracy of the early postnatal 25 and lifelong postnatal 50 Mn groups tended to be lower than controls in the 3 sec pre-cue delay trials of the focused attention task (*p* = 0.081 and 0.089, respectively), but not the baseline task (Figure 4B). This pattern of effects suggests that impaired arousal regulation likely contributed to the dysfunction seen in these Mn groups in the focused attention task as well.

### Behavioral Deficits Produced by Postnatal Mn Depend Upon the Duration of Exposure and its Interaction with Dose

The early postnatal (i.e., pre-weaning) exposure period is a particularly important window of Mn exposure susceptibility, since the same daily oral exposure over this period (normalized to body wt.) produced substantially higher blood and brain Mn levels compared to the same, albeit more prolonged, exposures during adolescence and adulthood (Table 1). Further, the pattern of attention and arousal regulation impairment across the early and lifelong postnatal Mn groups indicates that *a*) the early postnatal developmental window is particularly sensitive to the neurotoxic effects of oral Mn exposure, and *b*) the presence and nature of dysfunction depends somewhat on both the timing and duration of Mn exposure and its interaction with dose. For example, lifelong exposure to the higher 50 mg Mn/kg/day dose produced attentional deficits that were comparable to, or slightly more pronounced, than the deficits produced by early postnatal exposure alone. In contrast, the early postnatal 25 Mn group showed clear impairment in selective and focused attention, and arousal regulation, whereas their lifelong postnatal 25 Mn counterpart showed only a trend towards a selective attention dysfunction (first two session blocks of 2 sec distractor condition trials, Figure 2A,B), and no deficits in focused attention or arousal regulation. This suggests that lifelong postnatal exposure to the lower 25 Mn dose lessened the attention impairment caused by the early postnatal exposure to this same Mn dose. While non--monotonic dose–response relationships are well known in the toxicology and pharmacology literature, the mechanistic bases underlying these relationships are not well understood. As an essential metal capable of exerting positive and negative biological effects, possibly via anti-oxidant and pro-oxidant mechanisms that may vary with dose over the lifespan, the non-monotonic dose–response observed here is not unexpected. These observations may help explain the seeming disparity of results from the pediatric Mn studies, which have reported a range of associations between environmental or body Mn levels and neurobehavioral effects in cohorts where the Mn exposure history (timing, duration, magnitude) is typically not well known (Claus Henn et al. 2010; Lucchini et al. 2012; Oulhote et al. 2014; Takser et al. 2003).

### Possible Neurobiological Substrates Mediating the Mn-Induced Behavioral Dysfunction

These selective impairments in attentional function and arousal regulation due to postnatal Mn exposure implicate underlying dysfunction of both dopaminergic and noradrenergic systems of medial prefrontal cortex and subcortical structures that project to and/or receive projections from the prefrontal cortex (e.g., ventral tegmental area). It is well known that catecholaminergic systems in the prefrontal cortex are critical for the control of attentional processes, behavioral inhibition, and working memory, as well as arousal level and emotional self-regulation (Arnsten and Pliszka 2011; Arnsten 2009; Brennan and Arnsten 2008; Raz and Buhle 2006). Selective depletion of norepinephrine in the prefrontal cortex in rats produced deficits on a 5-CSRTT of focused and sustained attention (Carli et al. 1983; Milstein et al. 2007), similar to the pattern of deficits produced by early postnatal Mn exposure reported here. Further, we have reported previously that chronic postnatal Mn exposure reduced K^+^-stimulated dopamine and norepinephrine release in the prefrontal cortex and striatum of these same animals in adulthood (Beaudin et al. 2015), consistent with prior studies reporting reduced catecholamine neurotransmitter release and altered dopamine-1 (D1) and D2 receptor and dopamine transporter protein levels in the prefrontal cortex and striatum of rodents developmentally exposed to these same or similar oral doses of Mn (Beaudin et al. 2015; Kern and Smith 2011; Kern et al. 2010; McDougall et al. 2008; Reichel et al. 2006).

### Human Implications and Conclusions

Given that attentional function and arousal regulation affect many other cognitive functions (Bell and Deater-Deckard 2007; Raz and Buhle 2006), it can be expected that impairments in these areas will adversely affect academic performance and adaptive behavior. Our findings showing that early postnatal oral Mn exposure can cause lasting impairments in selective and focused attention and arousal regulation, without altering impulse control, are consistent with a predominantly ADHD-inattentive phenotype. Moreover, our prior studies have shown that the same animals tested here also displayed lasting impairment in fine motor function (Beaudin et al. 2013), findings that are consistent with the human literature indicating that children with attentional problems often perform poorly on motor skill tests (Kaiser et al. 2015). This pattern of Mn effects suggests lasting dysfunction of prefrontal catecholaminergic systems and supports suggestions that developmental Mn exposure may be an important risk factor for attentional dysfunction in children.

## Supplemental Material

(248 KB) PDF
