# Supplementary material for: Early Postnatal Manganese Exposure Causes Lasting Impairment of Selective and Focused Attention and Arousal Regulation in Adult Rats
Source: Environ Health Perspect. 2016 Jul 6;125(2):230–7. doi: 10.1289/EHP258 (PMC5289906; doi:10.1289/EHP258)
Supplement: (248 KB) PDF [file EHP258.s001.acco.pdf]

Environ Health Perspect
DOI: 10.1289/EHP258

Note to readers with disabilities: *EHP* strives to ensure that all journal content is accessible to all readers. However, some figures and Supplemental Material published in *EHP* articles may not conform to 508 standards due to the complexity of the information being presented. If you need assistance accessing journal content, please contact ehp508@niehs.nih.gov. Our staff will work with you to assess and meet your accessibility needs within 3 working days.

Supplemental Material

Early Postnatal Manganese Exposure Causes Lasting Impairment of Selective and Focused Attention and Arousal Regulation in Adult Rats

Stephane A. Beaudin, Barbara J. Strupp, Myla Strawderman, and Donald R. Smith

Table of Contents

1. Manganese exposure protocol and rationale
2. Behavioral testing procedures
3. Methods for determining blood and brain Mn concentrations
4. Animal body weights over the course of the study
   Figure S1. Body weights of rats in the five Mn exposure groups, as a function of postnatal age (days) in the study (n=21-23/group). Symbols at each PND age are treatment group mean body weights. Error bars are omitted for clarity. Manganese doses are in mg Mn/kg/d.
5. Behavioral testing results augmenting results provided in the main text
   Figure S2. Postnatal Mn exposure causes trending dose and duration-dependent deficits in the focused attention task. Percent correct responses for the early postnatal and lifelong postnatal Mn doses as a function of increasing pre-cue delay (n=21-23/group). † indicates $0.05 \leq p \leq 0.10$ for the lifelong 50 versus control; '&' indicates $0.05 \leq p \leq 0.10$ for the lifelong 50 versus lifelong 25 group. Manganese doses are in mg Mn/kg/d.

Figure S3. Postnatal Mn exposure causes dose and duration-dependent deficits in the selective attention task. Percent correct responses (%) for (A) the early postnatal Mn doses and (B) lifelong postnatal Mn doses, as a function of session block for each distraction condition (no odor, odor distractor 1 s or 2 s into the pre-cue delay interval) (n=21-23/group). * indicates $p \leq 0.05$ for the early 25 versus control in 'A', and lifelong 50 versus control in 'B'; † indicates $0.05 \leq p \leq 0.10$ for the early 25 or early 50 versus control in 'A', and lifelong 50 versus control in 'B'; '&' indicates $0.05 \leq p \leq 0.10$ between the two lifelong Mn groups in 'B'. Manganese doses are in mg Mn/kg/d.

6. References cited in Supplemental Material

Supplemental material

1. Manganese exposure protocol and rationale

Animals (dams and weaned pups) were fed Harlan Teklad rodent chow #2018 (reported by the manufacturer to contain 118 mg Mn/kg) and housed in polycarbonate cages at a constant temperature of 21 ± 2 ^{o}C. Neonates were orally exposed to 0, 25, or 50 mg Mn/kg/d beginning on PND 1. These Mn exposure levels are not overtly toxic and do not measurably affect neonate health or nutrition, based on neonate milk intake from the lactating dam, growth rate, or blood hematocrit levels at weaning. For Mn dosing over PND 1- 21, a 225 mg Mn/mL stock solution was prepared by dissolving $MnCl_2 \cdot 4H_2O$ with Milli-Q™ water; aliquots of the stock solution were diluted with a 2.5% (w/v) solution of the natural sweetener stevia for oral dosing of the neonates. Dosing solutions ranged from ~0.006 to 0.1 mg Mn/µL over the course of pre-weaning exposure as the animals gained body wt. Doses were delivered once a day directly into the mouth of the pups in a volume of ~25 µL/dose via a micropipette fitted with a flexible polyethylene gel loading tip (Fisher Scientific, Santa Clara, CA, USA). Control animals received only the deionized water + stevia vehicle. Daily oral Mn exposure post-weaning (PND 22 – end of study) was via the animal's drinking water. For this, a 42 mg Mn/mL stock solution was prepared fresh weekly as above and diluted with tap water in polycarbonate carboys to a final concentration of ~210 µg Mn/mL for the lifelong 25 mg Mn/kg/d exposed group, or ~420 µg Mn/mL for the 50 mg Mn/kg/d exposed group; actual water Mn levels were adjusted weekly if needed to maintain target exposure levels based on water intake. The stock solutions were made fresh weekly, and water bottles were refilled with fresh water two to three-times per week. Water bottle weights were recorded at refilling to determine Mn intake per cage, and daily Mn intake per kg body weight based on daily measured body weights of the two rats housed per cage.

The environmental relevance of the oral Mn dosing regimen used here is based on the following: The 25 and 50 mg Mn/kg/d exposure levels over the pre-weaning period produce relative increases in Mn intake that are ~350 and ~700-fold over levels consumed from lactation alone, which approximates the relative ~300 to 500-fold increases in Mn exposure experienced by infants and young children exposed to Mn-contaminated water or soy-based formulas (or both), compared to Mn ingestion from human breast milk (Kern et al. 2010). For example, human breast milk contains ~6 µg Mn/L, yielding normal infant intake rates of ~0.6 µg Mn/kg/d, based on infant daily milk consumption rates of ~0.8 L/day for a 8 kg 6-9 month old infant . Infants consuming contaminated water (e.g., directly or indirectly to rehydrate powdered formulas) containing 1.5 mg Mn/L, i.e., a level three-times the maximum contaminant level (MCL) guideline and comparable to median well-water levels associated with cognitive deficits and other effects in children, would experience Mn exposure of ~200 µg/kg/d, which is ~300-fold higher than the level of Mn intake from breast milk based on median fluid intake rates of ~1 L/d for infants <1 yr of age . Further, infants consuming Mn-contaminated well-water containing 1.5 mg Mn/L mixed with a high Mn soy formula containing up to 1.0 mg Mn/L (total = 2.5 mg Mn/L) would ingest ~300 µg Mn/kg/d, which is ~500-times than the Mn intake from breast milk, assuming the above median fluid intake rates for infants <1 yr of age. By comparison, rat milk Mn levels are ~200-300 µg Mn/L , and pre-weaning rats consume an average of 260 mL/kg/d over PND 1–21. Thus, pre-weaning control rats consume ~70 µg Mn/kg/d, which is ~100-times higher than normal human infant Mn intake from breast milk. Since normal daily dietary requirements for Mn are not known for either infant humans or rats (Keen et al. 1981; Ljung and Vahter 2007), we chose here an exposure regimen that models the relative increase in Mn intake

experienced by human infants exposed to contaminated well-water or soy formulas, compared to human breast milk.

Lifelong postnatal oral exposure to the same 25 or 50 mg Mn/kg/d was maintained post-weaning via drinking water, since children may continue to suffer chronic elevated Mn exposures from a variety of environmental sources (e.g., contaminated well-water, dust, etc.) (Bouchard et al. 2011; Lucas et al. 2015; Oulhote et al. 2014). Given that the Harlan Teklad rodent chow #2018 is reported to contain 118 mg Mn/kg, and that the adult rats were fed ~7% of body wt. per day (e.g., ~70 g/kg bw), adult rats consumed ~8.3 mg Mn/kg/d via the rodent chow. Thus, drinking water Mn intake to adults exposed to elevated Mn (i.e., 25 or 50 mg/kg/d) exceeded by ~3 – 6-fold Mn intake from the chow diet.

2. Behavioral testing procedures

Food magazine training: Each rat was first trained to retrieve the 45 mg food pellet rewards from the food magazine in the Med-Associates 5-CSRTT chambers. The first trial of a training session always began with a free-reward delivery and the illumination of the food magazine light and house light. A nose-poke to collect the free-reward extinguished the food magazine light. The house light remained illuminated. After 10 s had elapsed, the magazine light was illuminated again and another reward was delivered for the rat to collect. Magazine training continued in this fashion until each rat made 50 rewarded trials in a daily training session. No LEDs were illuminated on the opposite curved response wall during food magazine training.

Nose-poke training: Following food magazine training, each rat was trained to nose-poke into the five 2.5 x 2.5 cm response ports of the curved response wall of the chamber. Again, the first trial of a nose-poke training session always began with a free-reward delivery and the

illumination of the food magazine light and house light. A nose-poke into the food magazine to collect the free reward extinguished the food magazine light. The house light remained illuminated. In stage 1 of nose-poke training, a nose-poke into any one of the five response ports resulted in the immediate delivery of a food reward and the illumination of the food magazine light. Nose-poke training continued in this fashion over five consecutive stages that rewarded nose-pokes to ports 1, 2, 3, 4, and 5, respectively. This was done to ensure that all response ports were equally associated with a food reward before the start of testing proper. Each stage was completed after 70 rewarded trials. No response port LEDs were illuminated during nose-poke training.

Visual discrimination task: Following food magazine and nose-poke training, animals were trained in the 5-choice visual discrimination task: The first trials of each testing session also always began with a free-reward delivery and the illumination of the food magazine and house lights. After the rat interrupted the photo beam at the entrance of the food magazine (and extinguished the food magazine light), it was given 3 s to turn and reorient towards the opposite response wall, where one of the five response port LEDs was illuminated until the rat made a nose-poke into a port or after 10 s had elapsed, whichever came first. A nose-poke into the illuminated response port was tallied as a correct response and resulted in reward delivery and the illumination of the food magazine light. A nose-poke into a non-illuminated port was tallied as an incorrect response and was unrewarded and followed by a 5 s 'time-out' with the house light extinguished. Failure to make a nose-poke within the 10 s response interval (i.e., response omission) was also unrewarded and followed by a 5 s 'time-out' with the house light extinguished. The time-out was reset each time a response port nose-poke was recorded during the time-out period as a correction procedure. After the 5 s time-out, both the food magazine and

house lights were illuminated to signal the start of the next trial. Each rat was tested on the learning task to a criterion of ≥80% correct responses over two out of three consecutive testing sessions.

Response latency: Time latencies of nose-poke responses and latencies to collect food rewards following correct responses were also recorded on each trial in a session. Response latency was defined as the elapsed time between visual cue presentation and when the rat interrupted the photo beam at the entrance of one of the response ports. Food magazine latency was also recorded after each correct response and defined as the elapsed time between a response port nose-poke and when the rat interrupted the photo beam at the entrance of the food magazine.

Olfactory distractors in the selective attention task: The nine different liquid odorants used as olfactory distractors were prepared from pure liquid extracts of anise, maple, almond, peppermint, rum, orange, butter, cinnamon, and coconut (McCormick & Company, Inc., MD USA). Different concentrations of the pure liquid extracts ranging from 2.5% to 10% (v/v) were diluted with propylene glycol in a final volume of 200 mL liquid odorant. Twenty-five mL of the final solution was transferred via pipette into individual odor delivery jars mounted on the olfactory module on the outside wall of the sound attenuating cubicle housing the 5-CSRTT chamber. Odorized air was delivered for ~1 s into a response port within the chamber, with the condition that the visual cue response port and olfactory distractor response port never coincided within a trial. Delivery of odorized air used air pumps and computer-controlled solenoid valves for each chamber. Each response port contained an air inlet port and air evacuation port. During odor delivery, a vacuum pump fitted to the air evacuation port within the response port evacuated the odorized air so that it remained within the confines of the response port and did not enter the greater testing chamber space.

3. Methods for determining blood and brain Mn concentrations

Blood and brain Mn concentrations were determined in littermates of the study animals at PND 24 and PND 66 (7 – 8/treatment group and time point), and in the study animals at the completion of all behavioral testing (~PND 500). Animals were euthanized via sodium pentobarbital overdose (75 mg/kg ip) and exsanguination, and whole blood (2 – 3 mL) was collected from the left ventricle of the surgically-exposed heart and stored in EDTA Vacutainers at -20 ºC for analyses. Whole brain was immediately removed, bisected into hemispheres, and the hind-brain regions of each hemisphere collected and stored at -80 ºC for Mn concentration determinations (forebrain was dedicated to other outcome measures to be reported elsewhere). Briefly, aliquots of whole blood were digested overnight at room temperature with 16N HNO_3 (Optima grade, Fisher Scientific), followed by addition of H_2O_2 and Milli-Q™ water. Digestates were centrifuged (15,000 x g for 15 min.) and the supernatant collected for Mn analysis. For brain, aliquots of homogenized hind brain tissue (~200 mg wet weight) were dried then digested with hot 16 N HNO_3, evaporated and redissolved in 1 N HNO_3 for analyses. Rhodium was added to sample aliquots as an internal standard. Manganese levels were determined using a Thermo Element XR inductively coupled plasma – mass spectrometer, measuring masses ^{55}Mn and ^{103}Rh (the latter for internal standardization). External standardization for Mn used certified SPEX standards (Spex Industries, Inc., Edison, NJ). National Institutes of Standards and Technology SRM 1577b (bovine liver) was used to evaluate procedural accuracy. The analytical detection limit for Mn in blood and brain was 0.04 and 0.015 ng/mL, respectively.

4. Animal body weights over the course of the study.

All treatment groups gained body weight as expected over the course of the study (F(9, 989)=3404, p<0.0001), and there was no effect of Mn exposure or an interaction of Mn exposure and age on body weight (F(4, 104) = 0.70, p=0.59 and F(36, 989) = 0.97, p=0.51, respectively) (Supplemental Material, Figure S1).

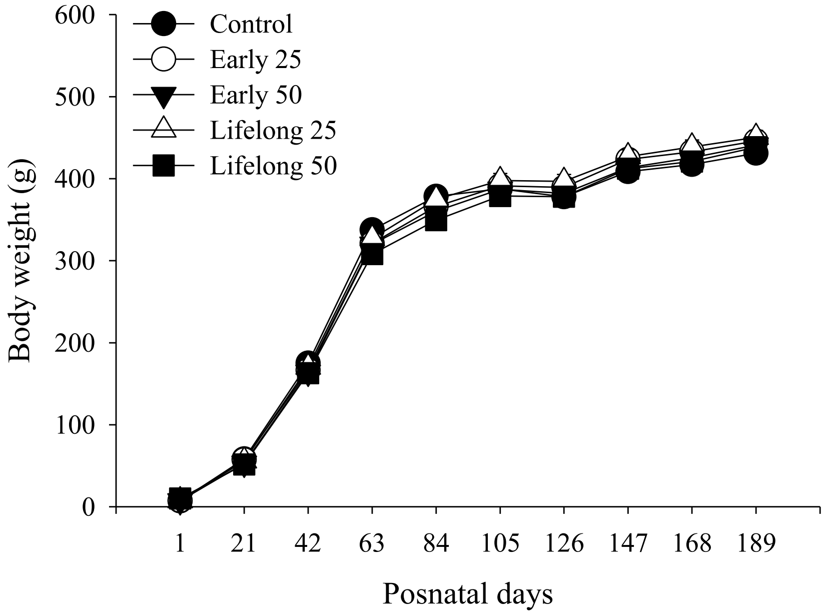

Figure S1. Body weights of rats in the five Mn exposure groups, as a function of postnatal age (days) in the study (n=21-23/group). Symbols at each PND age are treatment group mean body weights. Error bars are omitted for clarity. Manganese doses are in mg Mn/kg/d.

5. Behavioral testing results augmenting results provided in the main text.

Recorded response types for all behavioral tests were premature responses (responses made after trial onset but before presentation of the visual cue); correct response (responses made to the correct port following presentation of the visual cue); incorrect response (responses made to the incorrect port following presentation of the visual cue); and omissions (failure to respond within the 10 s response interval following the visual cue). As indicated in the main text,

response *accuracy* (i.e., percent accuracy) is defined as the percentage of the total "timely" responses (i.e., responses made within 10 s after visual cue presentation) made to the correct port, and is calculated as: % accuracy = [# correct responses / (correct + incorrect responses)] * 100. The '*percent correct*' '*percent incorrect*', '*percent prematures*', and '*percent omissions*' measures of performance are similar, but include all response types in the denominator, and are calculated as (showing % correct as an example): % Correct = [# correct responses / (correct + incorrect + omission + premature responses)] * 100.

5.1 Visual discrimination learning was not impaired by Mn

There was no effect of Mn treatment on measures of visual learning (trials and errors to criterion) in adult rats ($F(4, 103)=0.47$, $p=0.75$, and $F(4, 103)=0.66$, $p=0.71$, respectively) . On average, all of the treatment groups required 11.35 sessions or 1362 trials to achieve learning criterion on the visual discrimination task. There was also no effect of Mn exposure on omission errors (%) or on the mean latency to collect the food reward after a correct response (data not shown).

5.2 Focused attention task #1 (variable pre-cue delay, fixed cue duration) performance was not impaired by Mn

There was no significant main effect of Mn exposure on the percent correct responses or on percent accuracy ($F(4, 113)=0.31$, $p=0.73$, and $F(4, 113)=1.50$, $p=0.21$, respectively). Performance on these two outcome measures improved significantly across session block of testing, and decreased at the longer pre-cue delays, as expected (data not shown). However, the effect of session block or pre-cue delay on either performance measure did not differ between

any of the treatment groups (e.g., for percent accuracy $F(12, 193)=0.74$, $p=0.71$ and $F(16, 541)=1.39$, $p=0.14$, for session block and pre-cue delay, respectively). There was no significant main effect of Mn exposure on premature responses or omission errors (data not shown). However, premature responses increased and omission errors decreased with increasing pre-cue delay, as expected, but the effect of pre-cue delay was comparable across all treatment groups (e.g., for premature responses $F(8, 1497)=0.97$, $p=0.46$).

5.3 Focused attention task #2 (variable pre-cue delay, variable cue duration) performance was impaired by Mn

The main effect of Mn exposure was not significant for the percentage of correct responses ($F(4, 91)=0.89$, $p=0.47$) (Supplemental Material, Figure S2), similar to the results for percent accuracy reported in the main text $F(4, 103)=1.83$, $p=0.13$). There was, however, a significant main effect of pre-cue delay on both performance measures, as expected ($F(2, 223)=623.35$, $p<0.0001$ and $F(2, 542)=120.03$, $p<0.0001$ for percent correct and percent accuracy, respectively), with performance declining with increasing pre-cue delay (Supplemental Material, Figure S2 for percent correct responses). More importantly, there was a significant interaction of Mn exposure and pre-cue delay for both percent correct and percent accuracy, indicating that the effect of pre-cue delay on performance differed between Mn treatment groups, consistent with the findings reported in the main text (e.g., Mn X pre-due delay interaction $F(8, 391)=2.07$, $p=0.037$, and $F(8, 541)=2.59$, $p=0.009$, for percent correct and accurate responses, respectively). In contrast, no main effects of Mn exposure were seen on percent premature responses and percent omission errors, reflecting the significantly increasing prematures ($F(1, 110)=1460$, $p<0.0001$) and decreasing omission errors ($F(2, 831)=44.53$, $p<0.0001$) with

increasing pre-cue delay for all Mn groups. The effect of increasing pre-cue delay on prematures and omission errors in the focused attention task was comparable across all five Mn exposure groups (for prematures $F(4, 110)=1.37$, $p=0.25$, and for omissions $F(8, 831)=0.35$, $p=0.94$). Further, reducing the duration of the visual cue in the focused attention task also increased omission errors ($F(1, 535)=57.36$, $p<0.0001$), but to a comparable degree in all Mn groups (i.e., Mn exposure X cue duration interaction was $F(4, 535)=1.15$, $p=0.28$). Consistent with this pattern of results, there was a significant interaction of Mn exposure and pre-cue delay for the percent of incorrect responses ($F(8, 740)=3.28$, $p=0.001$), reflecting again that the effect of increasing pre-cue delay was significantly different between exposure groups. Finally, there was no significant effect of Mn exposure on latencies to collect food rewards or on the latencies of correct responses in the focused attention task (data not shown).

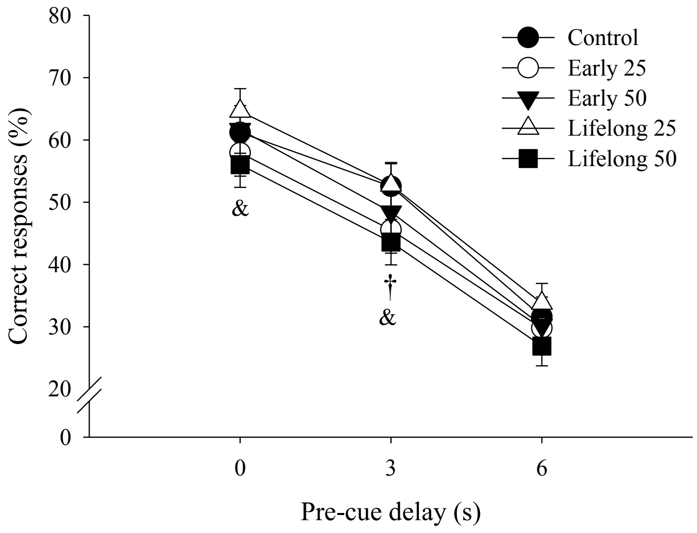

Figure S2. Postnatal Mn exposure causes trending dose and duration-dependent deficits in the focused attention task. Percent correct responses for the early postnatal and lifelong postnatal Mn doses as a function of increasing pre-cue delay (n=21-23/group). † indicates $0.05 \leq p \leq 0.10$ for

the lifelong 50 versus control; '&' indicates $0.05 \leq p \leq 0.10$ for the lifelong 50 versus lifelong 25 group. Manganese doses are in mg Mn/kg/d.

3.6. Selective attention task

Olfactory distracters significantly reduced the percentage of correct responses and response accuracy in all treatment groups, with the 2 s distraction trial condition (i.e., distractor presented 2 s into the trial, or 1 – 2 s prior to visual cue presentation) causing the greatest reduction in performance. Also, both performance measures improved significantly over consecutive session blocks of testing in all of the exposure groups, albeit to a lesser extent for distraction trials than non-distraction trials, as evidenced by an interaction between distractor condition and session block ($F(6, 2151)=24.09$, $p<0.0001$, and $F(6, 2315)=27.98$, $p<0.0001$ for percent correct and percent accuracy, respectively). There was no significant main effect of Mn exposure on correct or accurate responses ($F(4, 176)=1.56$, $p=0.19$, and $F(4, 130)=0.91$, $p=0.46$, respectively), though there was a significant interaction of Mn exposure X distractor X session block on correct responses (Supplemental Material, Figure S3), and a trending interaction with accuracy (main text Figure 2) ($F(24, 2151)=1.54$, $p=0.046$, and $F(24, 2315)=1.44$, $p=0.077$, for percent correct and accurate responses, respectively).

Presentation of olfactory distractors significantly increased the percentage of premature responses ($F(2, 253)=258.22$, $p<0.0001$), but did not alter omission error rates ($F(2, 403)=1.79$, $p=0.17$). However, there was no main effect of Mn exposure on premature responses or omission errors, nor an interaction of distractors X Mn exposure X session blocks for premature responses ($F(4, 200)=0.19$, $p=0.94$). Given this pattern of findings, analysis further uncovered a significant interaction of Mn exposure X distraction condition for the percent of incorrect responses (F(8,

529)=2.50, p=0.011), indicating that incorrect responses were significantly higher in almost all Mn exposure groups than controls specifically for the 2 s distraction condition (p's = 0.003, 0.046 and 0.018 for early 25 and 50, and lifelong 50 versus controls), with a similar trend seen in the lifelong 25 group (p=0.089). There was no effect of Mn exposure on latencies to collect food rewards or on the latencies of correct responses in the selective attention task (data not shown).

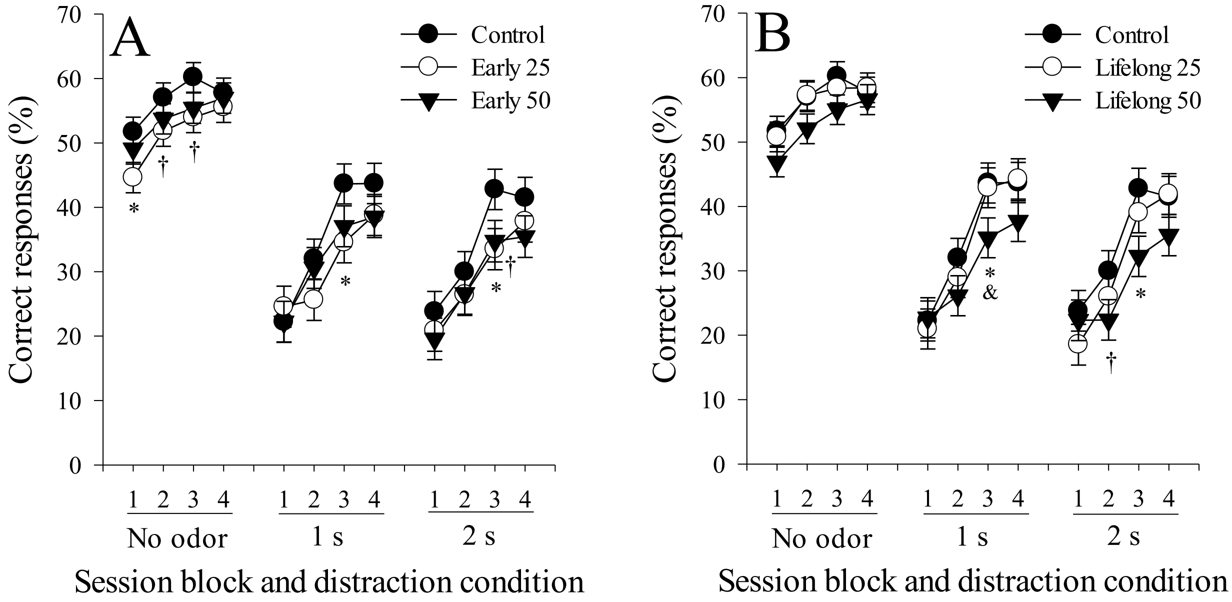

Figure S3. Postnatal Mn exposure causes dose and duration-dependent deficits in the selective attention task. Percent correct responses (%) for (A) the early postnatal Mn doses and (B) lifelong postnatal Mn doses, as a function of session block for each distraction condition (no odor, odor distractor 1 s or 2 s into the pre-cue delay interval) (n=21-23/group). * indicates $p \leq 0.05$ for the early 25 versus control in 'A', and lifelong 50 versus control in 'B'; † indicates $0.05 \leq p \leq 0.10$ for the early 25 or early 50 versus control in 'A', and lifelong 50 versus control in 'B'; '&' indicates $0.05 \leq p \leq 0.10$ between the two lifelong Mn groups in 'B'. Manganese doses are in mg Mn/kg/d.

6. References cited in Supplemental Material

Bouchard MF, Sauvé S, Barbeau B, Legrand M, Brodeur MÈ, Bouffard T, et al. 2011. Intellectual impairment in school-age children exposed to manganese from drinking water. Environ. Health Perspect. 119:138–143; doi:10.1289/ehp.1002321.

Keen CL, Lönnerdal B, Clegg M, Hurley LS. 1981. Developmental changes in composition of rat milk: trace elements, minerals, protein, carbohydrate and fat. J. Nutr. 111: 226–36.

Kern CH, Stanwood GD, Smith DR. 2010. Preweaning manganese exposure causes hyperactivity, disinhibition, and spatial learning and memory deficits associated with altered dopamine receptor and transporter levels. Synapse 64:363–378; doi:10.1002/syn.20736.

Ljung K, Vahter M. 2007. Time to re-evaluate the guideline value for manganese in drinking water? Environ. Health Perspect. 115:1533–1538; doi:10.1289/ehp.10316.

Lucas EL, Bertrand P, Guazzetti S, Donna F, Peli M, Jursa TP, et al. 2015. Impact of ferromanganese alloy plants on household dust manganese levels: Implications for childhood exposure. Environ. Res. 138:279–290; doi:10.1016/j.envres.2015.01.019.

Oulhote Y, Mergler D, Barbeau B, Bellinger DC, Bouffard T, Brodeur M-È, et al. 2014. Neurobehavioral Function in School-Age Children Exposed to Manganese in Drinking Water. Environ. Health Perspect. 122:1343–50; doi:10.1289/ehp.1307918.
